# Tardive dyskinesia among patients using antipsychotic medications in customary clinical care in the United States

**DOI:** 10.1371/journal.pone.0216044

**Published:** 2019-06-04

**Authors:** Anita M. Loughlin, Nancy Lin, Victor Abler, Benjamin Carroll

**Affiliations:** 1 Optum, Boston, Massachusetts, United States of America; 2 Teva Pharmaceuticals, Frazer, Pennsylvania, United States of America; University of Toronto, CANADA

## Abstract

**Background:**

Tardive dyskinesia (TD) is a movement disorder resulting from treatment with typical and atypical antipsychotics. An estimated 16–50% of patients treated with antipsychotics have TD, but this number may be underestimated. The objectives of this study were to build an algorithm for use in electronic health records (EHRs) for the detection and characterization of TD patients, and to estimate the prevalence of TD in a population of patients exposed to antipsychotic medications.

**Methods:**

This retrospective observational study included patients identified in the Optum EHR Database who received a new or refill prescription for an antipsychotic medication between January 2011 and December 2015 (follow-up through June 2016). TD mentions were identified in the natural language–processed clinical notes, and an algorithm was built to classify the likelihood that the mention represented documentation of a TD diagnosis as probable, possible, unlikely, or negative. The final TD population comprised a subgroup identified using this algorithm, with ≥1 probable TD mention (highly likely TD).

**Results:**

164,417 patients were identified for the antipsychotic population, with1,314 comprising the final TD population. Conservatively, the estimated average annual prevalence of TD in patients receiving antipsychotics was 0.8% of the antipsychotic user population. The average annual prevalence may be as high as 1.9% per antipsychotic user per year, allowing for a more-inclusive algorithm using both probable and possible TD. Most TD patients were prescribed atypical antipsychotics (1049/1314, 79.8%). Schizophrenia (601/1314, 45.7%), and paranoid and schizophrenia‐like disorders (277/1314, 21.1%) were more prevalent in the TD population compared with the entire antipsychotic drug cohort (13,308/164,417; 8.1% and 19,359/164,417; 11.8%, respectively).

**Conclusions:**

Despite a lower TD prevalence than previously estimated and the predominant use of atypical antipsychotics, identified TD patients appear to have a substantial comorbidity burden that requires special treatment and management consideration.

## Introduction

Tardive dyskinesia (TD) is a movement disorder that results from treatment with dopamine-receptor antagonists, including typical and atypical antipsychotics [1–3]. TD is characterized by involuntary, repetitive movements that can affect any part of the body, but predominantly affect the oro-buccal-lingual area [1, 4]. A majority of TD cases (87%) are irreversible, despite discontinuation of the causative drug [5]. TD symptoms can be socially stigmatizing and are associated with poor quality of life, increased morbidity and mortality [4]. To manage TD, clinicians typically reduce the dose of or discontinue the causative drug, which may result in relapse of the underlying psychiatric disorder and worsen the patient’s quality of life [1, 3, 6–8]. There are currently two vesicular monoamine transporter 2 (VMAT2) inhibitors approved by the US Food and Drug Administration for the treatment of TD, valbenazine and deutetrabenazine (both approved in 2017) [9, 10].

TD has an estimated lifetime prevalence of about 16–50% of patients treated with antipsychotics [3, 11, 12]. TD may not be apparent to patients and clinicians due to masking of symptoms by antipsychotics, patients' lack of awareness of dyskinesia symptoms, or physicians’ limited familiarity with TD [11, 13]. Masking of TD symptoms may occur due to the hypokinetic effects of antipsychotic medications, and may only become evident following treatment reduction, switching or discontinuation [14, 15]. More patients may be at risk for TD due to the expanded use of atypical antipsychotics to treat a variety of psychiatric conditions, including obsessive-compulsive disorder, eating disorders, and post-traumatic stress disorder [16–18]. In addition, other risk factors for TD, such as age, a pre-existing movement disorder, and exposure to other medication such as antiemetics, may increase a person’s risk for TD [19, 17]. Despite increased use of atypical antipsychotics, which vary in their likelihood to cause TD over typical antipsychotics, the burden of TD remains high [3, 20, 14, 21, 22]. To date, there are few literature reports that describe the clinical characteristics of patients with TD, despite its reported widespread prevalence.

The goals of this study were: 1) to identify and describe a population of patients with TD among an underlying population of patients receiving prescription antipsychotic medication; 2) to describe the underlying population, and 3) to estimate the prevalence of TD in this population.

## Methods

### Study design

This retrospective observational cohort study used electronic health record (EHR) data obtained from the Optum EHR Database between January 2010 and June 2016. The Optum EHR Database integrates EHRs from dozens of medical groups in the US, including over 195 hospitals, to form a broad patient-level database of healthcare encounters in everyday clinical practice. The database captures clinical, operational, and financial information recorded by physicians at the time of care. Within the underlying EHR systems, free-text information from inpatient and outpatient clinical notes of healthcare providers, as well as visit summaries, follow-up and referral letters, reports from imaging services, pathology investigations, surgical procedures, and other sources are available. Optum used a generalized natural language processing (NLP) system to extract and organize concepts from free-text into semi-structured data fields, along with pertinent sentiments (affirmations, and negations) and other modifiers (severity, duration and cause). Previously, Optum NLP algorithmic analysis of an EHR database has successfully provided clinical insights into hypoglycemia and binge-eating disorders [23–27]. In this study, descriptive characteristics of the underlying population of antipsychotic users and the population of patients with TD were assessed using data from EHR structured fields and data parsed from unstructured NLP clinical notes, which include such details as: demographics, healthcare utilization, antipsychotic prescription types, underlying psychiatric comorbidities, and the extrapyramidal symptoms. The average annual prevalence of TD among patients receiving antipsychotic medication was estimated.

### Ethics

The New England Institutional Review Board approved this project as an exempted retrospective study and determined that informed consent was not required.

### Study participants

Patients were included if they received a prescription (new or refill) for an antipsychotic between January 1, 2011 and December 31, 2015. A list of antipsychotics used in the study is provided in **S1 Table**. Patients entered the antipsychotic drug cohort on the date of the first antipsychotic prescription in the accrual period (anchor date) when all inclusion criteria were met. To be included in this analysis, the EHR had to contain clinical notes and the year of birth and the sex of the patient. The patients had to be at least 18 years old on the anchor date, have at least one outpatient clinic visit with an evaluation and management code during the 12‐month period prior to cohort entry (baseline period) excluding the anchor date, at least one outpatient visit or hospitalization 12 months–24 months prior to the anchor date, and at least one outpatient prescription during the baseline period excluding the anchor date.

### Antipsychotic drug cohort

Prevalent and new (treatment naïve) users of antipsychotics were eligible to enter the study cohort. A prevalent antipsychotic drug user was defined as a patient with a prescription for an antipsychotic, with evidence of previous prescription(s) for any antipsychotic drug (same or different) in the previous 12 months. A new antipsychotic drug user was defined as a patient with a prescription of antipsychotic drug on the anchor date, with no evidence of a prescription for any antipsychotic drug in the previous 12 months.

### TD population

Because there is no diagnostic code for TD, a subgroup of patients who were likely to have a diagnosis of TD was identified using an algorithm based on the mention of TD in the free-text clinical notes. The final algorithm focused on the single term “tardive dyskinesia” that was abstracted in the NLP clinical notes with the documentation of a sign, disease, or symptom in any section of the clinical note. Attributes, either sentiments or other modifiers, that provided context to each TD mention were reviewed and were used to classify the TD mention. TD mentions with sentiments indicating an affirmation (have, has, exhibit) were categorized as probable; clear negations (express not, deny, free) were categorized as negative; less clear fell into two categories, possible (seem, develop) or unlikely (concern, consider, describe). Supplementary modifiers were reviewed and modifiers that support a TD diagnosis (chronic, severe, longstanding, medication-induced, and controlled) or reference an affected body part (face, jaw, or lower extremity) could shift a TD mention initially classified as possible to probable. In addition, contextual information about medication orders was abstracted from the clinical notes; having TD listed as a reason for treatment was categorized as a probable TD mention. Hierarchically, according to their most likely TD mention, patients were classified as ‘highly likely TD’ if they had at least one probable TD mention. Patients were classified as ‘possibly likely TD’ if they had at least one possible TD mention. ‘Ambiguous unlikely TD’ cases had an unlikely TD mention, and ‘unlikely TD’ cases had negative TD mentions. Using structured fields, we excluded patients having a diagnosis of Parkinson’s disease (International Classification of Diseases [ICD]‐9: 332.0x, ICD‐10: G20.xx) or secondary parkinsonism (ICD‐9: 332.1, ICD‐10: G21.xx).

The final algorithm used to identify patients restricted the TD population to patients classified as highly likely TD. For each case, the date of earliest probable TD mention during the study period was considered the anchor date. Prior TD cases included patients with first probable TD mention in the baseline period, and new TD cases included patients with first probable TD mention during follow-up.

### Statistical analysis

The annual prevalence of TD was assessed among the underlying population of antipsychotic users available in each year of follow-up (2011–2016). To be included in the underlying population for a specific year (denominator), we required that the patient had met study eligibility criteria prior to or during the anchor year for prevalence estimation and had an observed outpatient medical encounter in the Optum EHR database during the anchor year or year prior. The requirement of an outpatient visit within 1 year of the anchor year for prevalence estimation provides some confidence that the patient continued to receive care that is documented in the Optum EHR database. Both prior and new TD cases were counted in the numerator of the prevalence calculation. In a specified year, a patient was included in the numerator if they were classified as a TD case during or prior to that year, and if the patient was included in the underlying population (denominator) for that year. Prevalence was calculated in two ways: 1) using a more restrictive definition that included highly likely TD cases only, and 2) using a more inclusive definition that included both highly likely and possible TD cases. For these measures, a summary estimate, average annual prevalence, is reported.

Baseline covariates of antipsychotic medication users and the subpopulation of patients with highly likely TD were derived from the structured fields in the EHR data during the 12-month baseline period. Characteristics included demographics and lifestyle characteristics, antipsychotic exposure, presence of a movement disorder, underlying comorbidities of interest, and mental health conditions. Prescription orders for antipsychotic medications were identified using National Drug Codes. Antipsychotic medications received on the anchor date were classified as either typical or atypical antipsychotic medications. Movement disorders, comorbidities and mental health conditions were identified using ICD-9-CM and ICD-10-CM diagnostic codes, as well as procedure codes and NLP data when appropriate. Descriptive analyses were performed to characterize the population taking antipsychotic medications and of the subpopulation of TD patients.

## Results

### Study population

**Fig 1:**
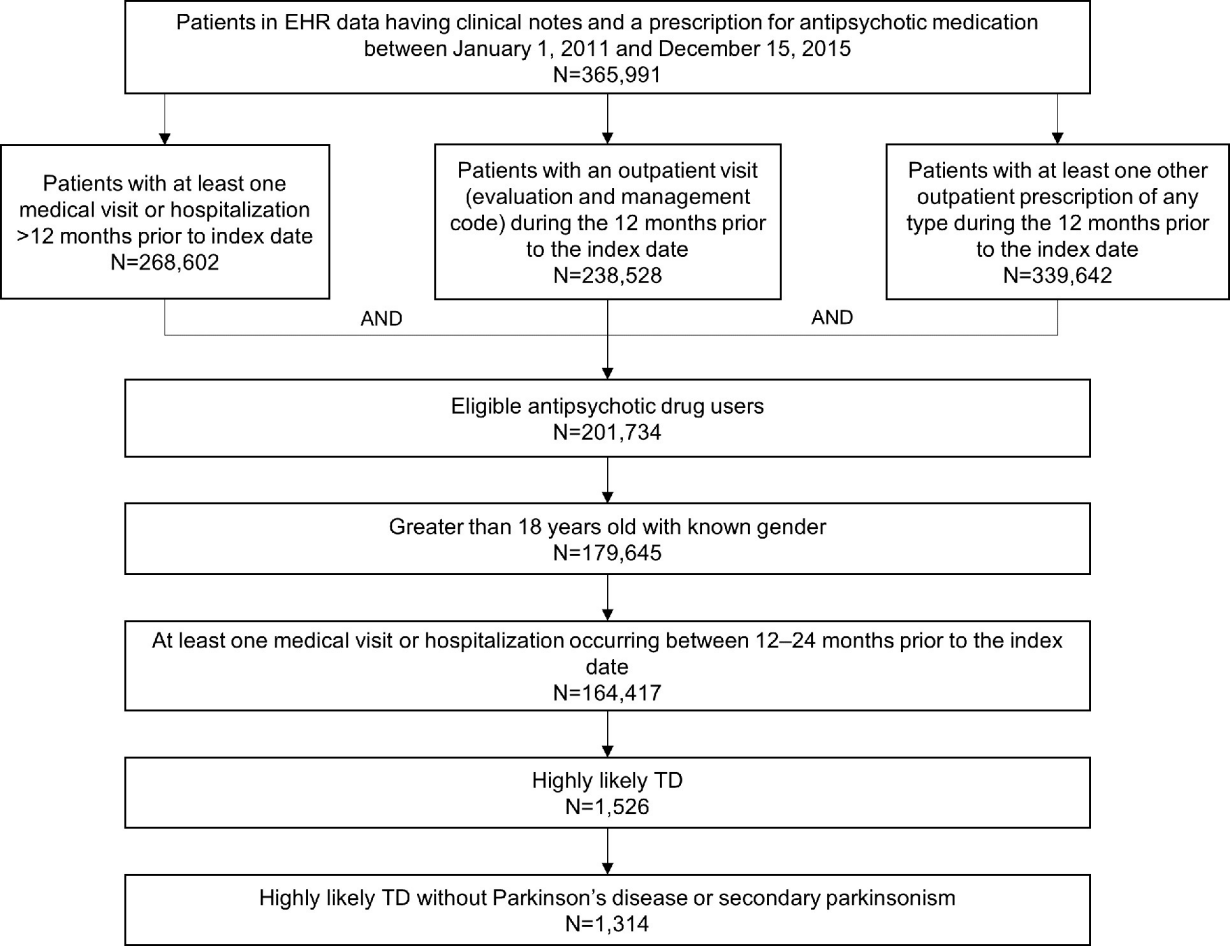
Study flow chart. EHR, electronic health record; TD, tardive dyskinesia.

A total of 164,417 patients were identified as having prescriptions for antipsychotics and ≥1 medical visit or hospitalization 12–24 months before the anchor date; in this population there were 6,294 (3.8%) patients with a mention of TD in their NLP clinical notes, and 1,314 (0.8%) that comprised the final TD population (**Fig 1**). The antipsychotic population was predominantly 50+ years of age (97,546/164,417; 59.3%), female (100,923/164,417; 61.4%), white (131,780/164,417; 80.2%), and had a diagnosis of a mood disorder (75,672/164,417; 46.0%) and/or neurotic/anxiety disorder (73,869/164,417; 44.9%) (**Table 1**). The final TD population consisted of 530 (40.3%) prior TD patients and 784 (59.7%) new TD patients.

**Table 1:** Frequency of baseline covariates among antipsychotic drug users.

Characteristic	Prevalent APD Users (N = 44,710) n (%)	New APD Users (N = 119,707) n (%)	All APD Users (N = 164,417) n (%)
**Age**			
<20 years	1,316 (2.9)	2,473 (2.1)	3,789 (2.3)
20–29 years	5,090 (11.4)	11,256 (9.4)	16,346 (9.9)
30–39 years	5,991 (13.4)	13,844 (11.6)	19,835 (12.1)
40–49 years	8,305 (18.6)	18,596 (15.5)	26,901 (16.4)
50–59 years	10,000 (22.4)	22,886 (19.1)	32,886 (20.0)
60–69 years	6,361 (14.2)	16,922 (14.1)	23,283 (14.2)
≥70 years	7,647 (17.1)	33,730 (28.2)	41,377 (25.2)
**Sex**			
Male	18,198 (40.7)	45,296 (37.8)	63,494 (38.6)
Female	26,512 (59.3)	74,411 (62.2)	100,923 (61.4)
**Race/ethnicity**			
White	34,206 (76.5)	97,574 (81.5)	131,780 (80.2)
Black/African American	5,144 (11.5)	10,398 (8.7)	15,542 (9.5)
Other	5,360 (12.0)	11,735 (9.8)	17,095 (10.4)
**Any Alcohol use/abuse**	3,529 (7.9)	8,981 (7.5)	12,510 (7.6)
**Ever a smoker**	35,694 (79.8)	106,091 (88.6)	141,785 (86.2)
**Underlying mental health condition**			
Dementia	6,278 (14.0)	25,706 (21.5)	31,984 (19.5)
Schizophrenia	6,841 (15.3)	6,467 (5.4)	13,308 (8.1)
Mood disorder	21,918 (49.0)	53,754 (44.9)	75,672 (46.0)
Paranoid and schizophrenia-like disorder	5,694 (12.7)	13,665 (11.4)	19,359 (11.8)
Neurotic and anxiety disorder	19,030 (42.6)	54,839 (45.8)	73,869 (44.9)
Personality disorder	2,253 (5.0)	4,278 (3.6)	6,531 (4.0)
Alcohol dependence	2,125 (4.8)	5,715 (4.8)	7,840 (4.8)
Drug dependence	11,189 (25.0)	30,987 (25.9)	42,176 (25.7)
**Comorbid movement disorders**			
Drug-induced subacute dyskinesia	188 (0.4)	251 (0.2)	439 (0.3)
Drug-induced acute dystonia or akathisia	206 (0.5)	520 (0.4)	726 (0.4)
Blepharospasms	26 (0.1)	74 (0.1)	100 (0.1)
Dyskinesia/dystonia	435 (1.0)	2,310 (1.9)	2,745 (1.7)
Orofacial dyskinesia	165 (0.4)	99 (0.1)	264 (0.2)
Major dystonia	57 (0.1)	94 (0.1)	151 (0.1)
Parkinson's disease	841 (1.9)	3,155 (2.6)	3,996 (2.4)
Secondary Parkinson's disease	175 (0.4)	318 (0.3)	493 (0.3)
Degenerative basal ganglia disease	719 (1.6)	2,507 (2.1)	3,226 (2.0)
Extrapyramidal movement disorder	1,596 (3.6)	5,171 (4.3)	6,767 (4.1)
**Underlying comorbidity**			
Diabetes	7,882 (17.6)	25,650 (21.4)	33,532 (20.4)
Dysphagia	1,324 (3.0)	7,489 (6.3)	8,813 (5.4)
Pneumonia	1,899 (4.3)	9,599 (8.0)	11,498 (7.0)
Alcoholism	3,333 (7.5)	8,607 (7.2)	11,940 (7.3)
Dyslipidemia	15,753 (35.2)	51,971 (43.4)	67,724 (41.2)
Obesity	28,667 (64.1)	82,391 (68.8)	111,058 (67.6)
Any falls	2,367 (5.3)	11,511 (9.6)	13,878 (8.4)
Any fractures	3,777 (8.5)	15,501 (13.0)	19,278 (11.7)
Any pain	19,258 (43.1)	68,250 (57.0)	87,508 (53.2)
**Healthcare resource utilization**			
Number of outpatient physician visits			
1–2	8,816 (19.7)	13,800 (11.5)	22,616 (13.8)
3–4	8,716 (19.5)	20,468 (17.1)	29,184 (17.8)
5–6	6,394 (14,3)	17,499 (14.6)	23,893 (14.5)
7+	20,784 (46.5)	67,940 (56.8)	88,724 (54.0)
Number of emergency department visits			
None	30,321 (67.8)	67,479 (56.4)	97,800 (59.5)
1–2	9,053 (20.3)	32,119 (26.8)	41,172 (25.0)
3–4	2,779 (6.2)	11,126 (9.3)	13,905 (8.5)
5–6	1,118 (2.5)	4,374 (3.7)	5,492 (3.3)
7+	1,439 (3.2)	4,609 (3.9)	6,048 (3.7)
Number of inpatient stays			
None	35,665 (79.8)	80,655 (67.4)	116,320 (70.8)
1–2	6,320 (14.1)	26,424 (22.1)	32,744 (19.9)
3–4	834 (1.9)	3,560 (3.0)	4,394 (2.7)
5–6	392 (0.9)	1,746 (1.5)	2,138 (1.3)
7+	1,499 (3.4)	7,322 (6.1)	8,821 (5.4)

APD, antipsychotic drug; TD, tardive dyskinesia.

### Estimated TD prevalence

Based on highly likely TD cases, the annual prevalence of TD in patients receiving antipsychotics ranged from 7.6–9.7 per 1000 patients during the study period. The average annual prevalence estimate of probable TD was 7.8 per 1000 patients during the interim years (0.8% of the antipsychotic user population per year). Counting both highly likely and possibly likely TD cases, the annual prevalence of probable or possible TD among patients receiving antipsychotics ranged from 18.0 to 20.5 per 1000 patients during the study period. The average annual prevalence estimate of probable or possible TD was 18.8 per 1000 patients during the interim years (1.9% of the antipsychotic user population per year).

### Study population characteristics

Similarly to the overall study population, the majority of the TD population were 50 years of age or older (952/1314; 72.4%), female (800/1314; 60.9%), and white (881/1314; 67.1%) (**Table 2**). While patients could have codes for more than one mental health condition, approximately half of the TD population had evidence of a neurotic/anxiety disorder (693/1314; 52.7%), or a mood disorder (661/1314; 50.3%) (**Table 2**). Few of the patients prescribed an antipsychotic drug or who had TD had evidence of alcohol use, abuse, or dependence, but most had a history of smoking and about one-quarter to one‐third had evidence of drug dependence, respectively (**Tables 1** and **2**).

**Table 2:** Frequency of baseline covariates among antipsychotic drug users with TD.

Characteristic	Prior TD (N = 530) n (%)	New TD (N = 784) n (%)	All TD (N = 1314) n (%)
**Age**			
20–29 years	16 (3.0)	40 (5.1)	56 (4.3)
30–39 years	37 (7.0)	56 (7.1)	93 (7.1)
40–49 years	98 (18.5)	112 (14.3)	210 (16.0)
50–59 years	150 (28.3)	216 (27.6)	366 (27.9)
60–69 years	124 (23.4)	206 (26.3)	330 (25.1)
≥70 years	105 (19.8)	151 (19.3)	256 (19.5)
**Sex**			
Male	234 (44.2)	280 (35.7)	514 (39.1)
Female	296 (55.9)	504 (64.3)	800 (60.9)
**Race/ethnicity**			
White	299 (56.4)	582 (74.2)	881 (67.1)
Black/African American	197 (37.2)	135 (17.2)	332 (25.3)
Other	34 (6.4)	67 (8.5)	101 (7.7)
**Any alcohol use/abuse**	67 (12.6)	80 (10.2)	147 (11.2)
**Ever a smoker**	505 (95.3)	690 (88.0)	1195 (90.9)
**Underlying mental health condition**			
Dementia	107 (20.2)	116 (14.8)	223 (17.0)
Schizophrenia	325 (61.3)	276 (35.2)	601 (45.7)
Mood disorder	250 (47.2)	411 (52.4)	661 (50.3)
Paranoid and schizophrenia-like disorder	122 (23.0)	155 (19.8)	277 (21.1)
Neurotic and anxiety disorder	324 (61.1)	369 (47.1)	693 (52.7)
Personality disorder	51 (9.6)	65 (8.3)	116 (8.8)
Alcohol dependence	43 (8.1)	52 (6.6)	95 (7.2)
Drug dependence	202 (38.1)	243 (31.0)	445 (33.9)
**Comorbid movement disorders**			
Drug-induced subacute dyskinesia	183 (34.5)	39 (5.0)	222 (16.9)
Drug-induced acute dystonia or akathisia	13 (2.5)	10 (1.3)	23 (1.8)
Blepharospasms	1 (0.2)	1 (0.1)	2 (0.2)
Dyskinesia/dystonia	19 (3.6)	18 (2.3)	37(2.8)
Orofacial dyskinesia	93 (17.6)	20 (2.6)	113 (8.6)
Major dystonia	3 (0.6)	3 (0.4)	6 (0.5)
Parkinson's disease	0	0	0
Secondary Parkinson's disease	0	0	0
Degenerative basal ganglia disease	20 (3.8)	22 (2.8)	42 (3.2)
Extrapyramidal movement disorder	256 (48.3)	97 (12.4)	353 (26.9)
**Underlying comorbidity**			
Diabetes	164 (30.9)	216 (27.6)	380 (28.9)
Dysphagia	37 (7.0)	45 (5.7)	82 (6.2)
Pneumonia	37 (7.0)	54 (6.9)	91 (6.9)
Alcoholism	67 (12.6)	80 (10.2)	147 (11.2)
Dyslipidemia	258 (48.7)	377 (48.1)	635 (48.3)
Obesity	388 (73.2)	580 (74.0)	968 (73.7)
Any falls	61 (11.5)	62 (7.9)	123 (9.4)
Any fractures	76 (14.3)	108 (13.8)	184 (14.0)
Any pain	254 (47.9)	372 (47.5)	626 (47.6)
**Healthcare resource utilization**			
Number of outpatient physician visits			
1–2	37 (7.0)	80 (10.2)	117 (8.9)
3–4	57 (10.8)	116 (14.8)	173 (13.2)
5–6	55 (10.4)	93 (11.9)	148 (11.3)
7+	381 (71.9)	495 (63.1)	876 (66.7)
Number of emergency department visits			
None	249 (47.0)	414 (52.8)	663 (50.5)
1–2	140 (26.4)	200 (25.5)	340 (25.9)
3–4	61 (11.5)	74 (9.4)	135 (10.3)
5–6	36 (6.8)	36 (4.6)	72 (5.5)
7+	44 (8.3)	60 (7.7)	104 (7.9)
Number of inpatient stays			
None	301 (56.8)	523 (66.7)	824 (62.7)
1–2	136 (25.7)	154 (19.6)	290 (22.1)
3–4	21 (4.0)	26 (3.3)	47 (3.6)
5–6	13 (2.5)	14 (1.8)	27 (2.1)
7+	59 (11.1)	67 (8.6)	126 (9.6)

TD, tardive dyskinesia.

### Comorbidities

**Fig 2:**
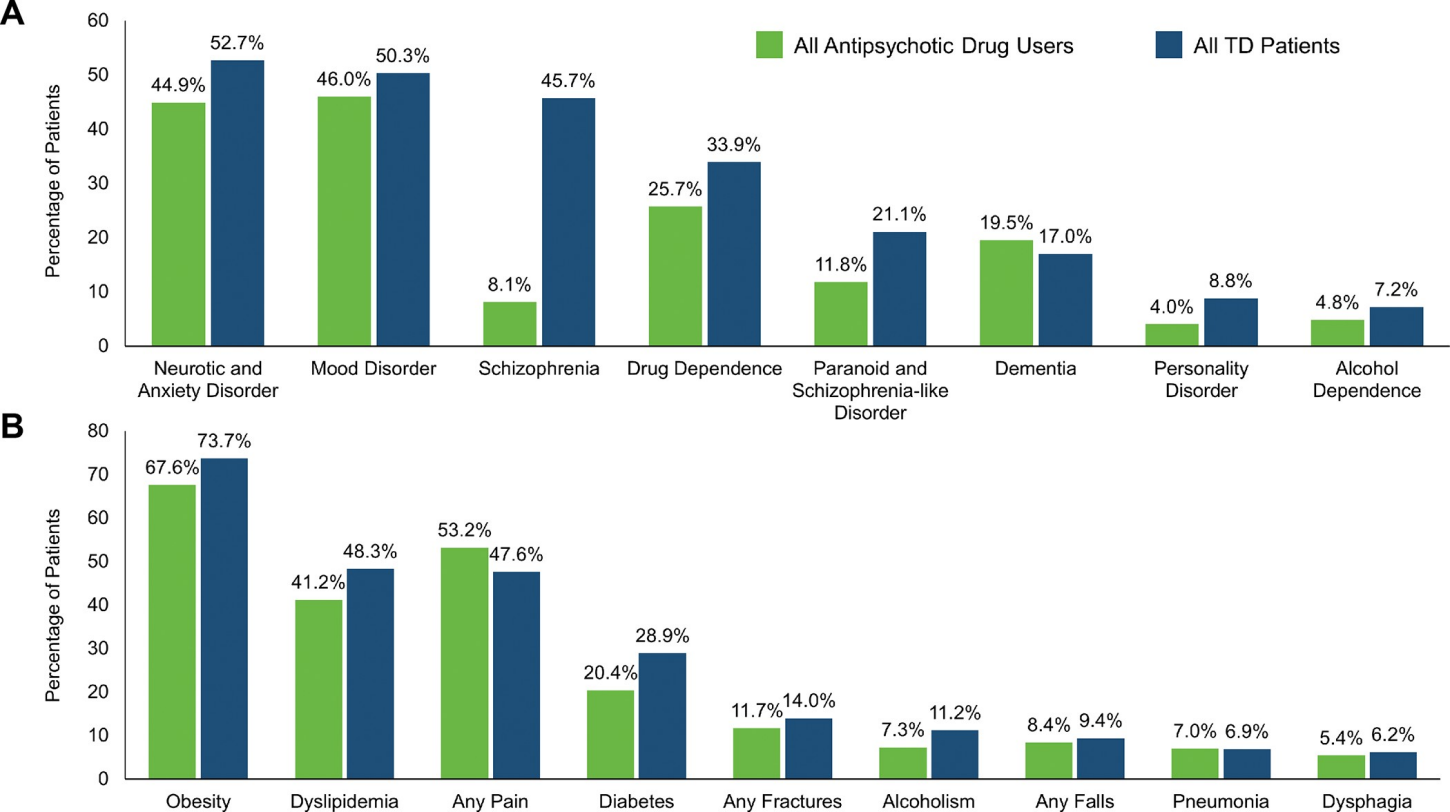
**(A) Underlying mental health conditions and (B) underlying disease comorbidities in antipsychotic drug users compared with tardive dyskinesia patients.** TD, tardive dyskinesia.

Schizophrenia (601/1314; 45.7%), and paranoid and schizophrenia‐like disorders (277/1314; 21.1%) were more prevalent in the TD population than in the entire antipsychotic drug cohort (13,308/164,417 [8.1%] and 19,359/164,417 [11.8%], respectively) (**Fig 2**).

Approximately two‐thirds of the TD population had a structured code for obesity (929/1314; 70.7%). Fewer patients had evidence of a diabetes diagnosis (390/1314; 29.7%) or dyslipidemia (589/1314; 44.8%). Underlying comorbidities of obesity, diabetes, and dyslipidemia were more prevalent in the TD population than in the entire antipsychotic drug cohort (**Fig 2**).

### Antipsychotic use

More than half of the TD population were classified as prevalent antipsychotic users (716/1314; 54.5%). Most TD patients were prescribed atypical antipsychotics (1049/1314; 79.8%) and received only one prescription for an antipsychotic drug (1197/1314; 91.1%). The most commonly prescribed atypical antipsychotics were quetiapine (546/1314; 41.6%), risperidone (412/1314; 31.4%), aripiprazole (322/1314; 24.5%), and olanzapine (298/1314; 22.7%). Between baseline and proximal to the first probable TD mention (2 months prior to 6 months after the date of first probable TD mention), more than half of the patients in the new TD population (451/784; 57.5%) had an apparent switch in their antipsychotic medication from their index antipsychotic and 12/784 patients (1.5%) had their dose lowered.

### Movement disorders

**Fig 3:**
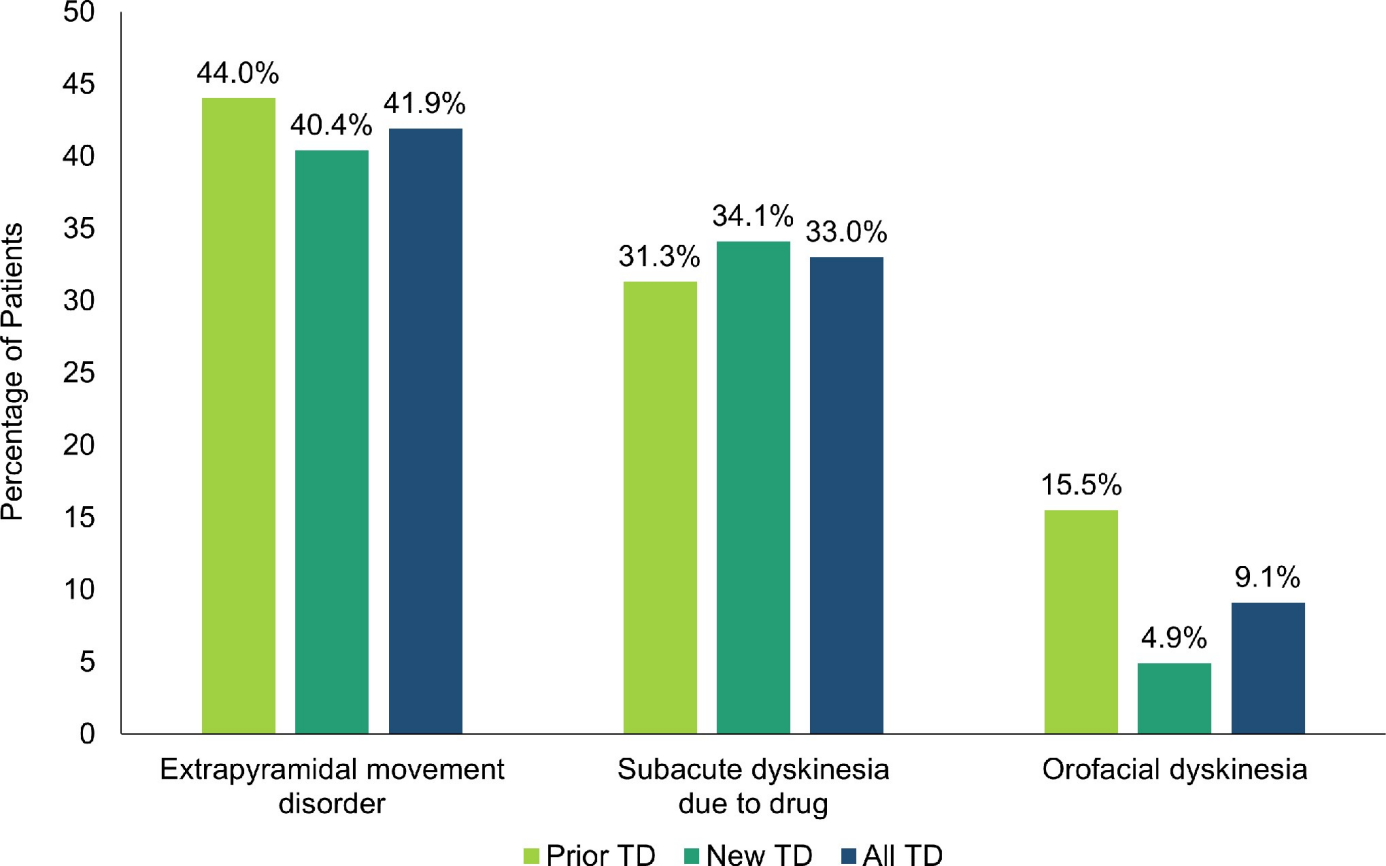
Most common movement disorders proximal to the first mention of probable tardive dyskinesia. TD, tardive dyskinesia.

The TD population had greater baseline occurrence of movement disorders than the antipsychotic population (**Tables 1** and **2**), including extrapyramidal movement disorder (all TD: 353/1314 [26.9%]; all antipsychotic: 6767/164,417 [4.1%]), drug-induced subacute dyskinesia (all TD: 222/1314 [16.9%]; all antipsychotic: 439/164,417 [0.3%]), and orofacial dyskinesia (all TD: 113/1314 [8.6%]; all antipsychotic users: 264/164,417 [0.2%]). The frequency of movement disorders among new TD patients was similar to that measured among prior TD patients. Proximal to the first probable TD mention, the three most common movement disorders were extrapyramidal movement disorder (550/1314; 41.9%), drug-induced subacute dyskinesia (33/1314; 33.0%), and orofacial dyskinesia (prior TD: 82/530 [15.5%]; new TD: 38/784 [4.9%]; all TD: 120/1314 [9.1%)] (**Fig 3**).

### Healthcare utilization

A majority of the TD population had 3 or more outpatient visits during the baseline period (1197/1314; 91.1%) compared with 86.2% (141,801/164,417) in the antipsychotic population (**Tables 1** and **2**). About one-half of the TD population had at least one visit to the emergency department (651/1314; 49.5%]) and more than one‐third had at least one inpatient stay during baseline period (490/1314; 37.3%). Emergency department and inpatient stays were less prevalent in the antipsychotic population (66,617/164,417 [40.5%] and 48,097/164,417 [29.3%], respectively) than in the TD population.

## Discussion

In this retrospective, descriptive analysis, we used natural language processing to extract information from clinical notes and built an algorithm to identify probable cases of TD. This information was used to evaluate the clinical characteristics of prevalent and new antipsychotics users, including those deemed highly likely to have a TD diagnosis.

Consistent with previous studies, we found that in clinical practice a majority of patients in the TD population received atypical rather than typical antipsychotics [14, 21, 22]. Despite the use of these newer antipsychotics, the burden of TD within this population persisted. We estimated a TD prevalence of 0.8%–1.9% of people taking antipsychotics, depending on the stringency of our TD definition. This estimation is much lower than the previously published prevalence of 16–50% of patients taking antipsychotics [3, 11, 12]. However, it is important to note that previous prevalence estimates of TD may be affected by study populations that are reflective of the US population, or by studies that were conducted before atypical antipsychotics were widely used. One previous prevalence study estimated a TD prevalence of 31.5% of people taking antipsychotics; however, the study was conducted at only one health center and had a study population of 619 people [12]. The prevalence of TD estimated in the current study represents patient data taken from a large and geographically diverse number of medical practices in the US, in which over 160,000 antipsychotics users were identified. This prevalence estimate may reflect a true reduction in TD with the rise in use of atypical antipsychotics, may be due in part to under-documentation of TD cases in clinical notes, or may result from misclassification or incomplete capture of TD cases using the current algorithm.

Patients in the TD population had a quantitatively higher psychiatric comorbidity burden compared with all patients treated with antipsychotics. Because the algorithm favors specificity over inclusiveness when identifying TD diagnosis in the EHR database, the comparison between the highly likely TD group and the APD users group may have omitted a segment of the TD patient population. Furthermore, the substantial number of health records analyzed and the large absolute differences observed in these burden effects obviate the need for statistical analysis for the comparison of these groups. Therefore, these qualitative comparisons support the case for a real-world burden of TD in the clinical setting and demonstrate a novel means to extract burden from existing clinical data resources.

The TD population had a relatively higher prevalence of schizophrenia (absolute difference of 37.6%) compared with the antipsychotic population. In our analysis, psychiatric comorbidities appeared at a higher rate in the TD population relative to the antipsychotic population, including paranoid and schizophrenia-like disorder (9.3% difference), drug dependence (8.2% difference), neurotic/anxiety disorder (7.8% difference), personality disorder (4.8% difference), mood disorder (4.3% difference), and alcohol dependence (2.4% difference). Our data suggest the TD population had an increased non-psychiatric comorbidity compared with the general antipsychotic population, including diabetes (8.5% difference), dyslipidemia (7.1% difference), obesity (6.1% difference), alcoholism (3.9%), fractures (2.3% difference), falls (1.0% difference), and dysphagia (0.8% difference). Notably, the TD population had overall higher baseline occurrences of movement disorders, such as, extrapyramidal movement disorder (22.8% difference), drug-induced subacute dyskinesia (16.6% difference), and orofacial dyskinesia (8.4% difference).

In addition to the increased health burden, the TD population experienced a numerically larger healthcare burden than the general antipsychotic population, with increases in outpatient, inpatient, and emergency department visits. Future studies with an algorithm optimized for sensitivity will enable rigorous statistical evaluation of these trends. Reasons for the increased rates of comorbidities and increased healthcare utilization should also be explored in future studies.

This study was based on an analysis of EHR data. While EHR data are valuable for examination of clinical health care outcomes and treatment patterns, all EHR databases have certain inherent limitations because the data are collected for the purpose of clinical patient management, not research. The Optum EHR is not a closed system. Some patients may only receive a fraction of their care through a healthcare provider captured in the database, and medical encounters outside of the networks contributing data to the EHR will not be observed. In this study, the antipsychotic user cohort was restricted to patients who had clinical notes as part of their EHR; however, clinical notes may be incomplete if the patient sought care outside of the healthcare provider networks contributing to the Optum EHR database. To mitigate the potential for missing data, eligibility requirements were implemented to restrict the study population to patients with evidence of routine care within the contributing EHR systems.

Within the baseline period, we required that the patient have a recorded encounter in the EHR database at least 1 year prior to the initiation date, and at least one encounter with an outpatient evaluation and management procedure code within 1 year prior to the initiation date. Patients meeting these criteria likely have a reasonably high capture of medical encounters. In addition, we restricted the denominators for prevalence estimates to the periods of time when there was evidence that the patients had received continuous care. This study relies on information extracted from free-text clinical notes using a generalized NLP approach to identify and classify cases of TD; therefore, misclassification of the diagnosis of TD is possible. Clinical notes may include mentions of TD that are not indicative of a positive presence of TD. Opting for specificity over inclusiveness, the final algorithm included only patients with strongly affirmed TD mentions only (highly likely TD), and mentions that were initially classified as possible, but had supporting/affirming modifying information. The algorithm can be subsequently benchmarked using a set of patient records individually categorized as TD or non-TD diagnoses, and adjudicated by physician review to refine the algorithm if needed.

Despite these limitations, this study is one of the largest epidemiological studies of TD to date. Based on an algorithm used to extract information from EHR clinical notes, we found a lower estimated TD prevalence than previously published estimates. Although further work is needed to confirm these findings, understanding the characteristics of the TD patient population, specifically its substantial comorbidity and healthcare burden, informs healthcare providers responsible for the treatment and management of TD patients.

## Supporting information

S1 Table Antipsychotic medications identified for use in the study. Prescriptions orders for antipsychotic medications were identified using National Drug Codes in structured EHR data fields and classified by type (typical versus atypical).
(DOCX)
