# Supplementary material for: Tardive dyskinesia among patients using antipsychotic medications in customary clinical care in the United States
Source: PLoS One. 2019 Jun 4;14(6):e0216044. doi: 10.1371/journal.pone.0216044 (PMC6548364; doi:10.1371/journal.pone.0216044)
Supplement: S1 Table — Prescriptions orders for antipsychotic medications were identified using National Drug Codes in structured EHR data fields and classified by type (typical versus atypical). (DOCX) [file pone.0216044.s001.docx]

**S1 Table. Antipsychotic medications identified for use in the study.**

Prescriptions orders for antipsychotic medications were identified using National Drug Codes in structured EHR data fields and classified by type (typical versus atypical).

| **Antipsychotic Medications** |
| --- |
| **Typical Antipsychotic Medications** |
| Chlorpromazine HCl |
| Fluphenazine (fluphenazine decanoate, fluphenazine enanthate, fluphenazine HCl) |
| Haloperidol (haloperidol decanoate, haloperidol lactate, haloperidol) |
| Loxapine (loxapine, loxapine succinate, loxapine HCl) |
| Mesoridazine besylate |
| Molindone HCl |
| Perphenazine (perphenazine, perphenazine/amitriptyline HCl) |
| Pimozide |
| Promazine HCl |
| Thioridazine HCl |
| Thiothixene (thiothixene HCl) |
| Trifluoperazine HCl |
| **Atypical Antipsychotic Medications** |
| Aripiprazole (aripiprazole, aripiprazole lauroxil) |
| Asenapine |
| Brexpiprazole |
| Cariprazine HCl |
| Clozapine |
| Iloperidone |
| Lurasidone HCl |
| Olanzapine (olanzapine, olanzapine pamoate, olanzapine/fluoxetine HCl) |
| Paliperidone (paliperidone, paliperidone palmitate) |
| Pimavanserin (pimavanserin, pimavanserin tartrate) |
| Quetiapine fumarate |
| Risperidone (risperidone, risperidone microspheres) |
| Ziprasidone (ziprasidone HCl, ziprasidone mesylate) |

EHR, electronic health record.
